# *SOX6* and *PDCD4* enhance cardiomyocyte apoptosis through LPS-induced miR-499 inhibition

**DOI:** 10.1007/s10495-015-1201-6

**Published:** 2015-12-10

**Authors:** Zhuqing Jia, Jiaji Wang, Qiong Shi, Siyu Liu, Weiping Wang, Yuyao Tian, Qin Lu, Ping Chen, Kangtao Ma, Chunyan Zhou

**Affiliations:** Department of Biochemistry and Molecular Biology, School of Basic Medical Sciences, Beijing Key Laboratory of Protein Posttranslational Modifications and Cell Function, Key Laboratory of Molecular Cardiovascular Sciences, Ministry of Education of China, Peking University, No. 38, Xueyuan Road, Haidian District, Beijing, China; Beijing Jianhua Experimental School, Yuquan Road 66, Haidian District, Beijing, China; Department of Epidemiology, Rollins School of Public Health, Emory University, 1518 Clifton Road NE, Atlanta, GA 30322 USA

**Keywords:** *SOX6*, *PDCD4*, LPS, miR-499, Cardiomyocyte, Apoptosis

## Abstract

Sepsis-induced cardiac apoptosis is one of the major pathogenic factors in myocardial dysfunction. As it enhances numerous proinflammatory factors, lipopolysaccharide (LPS) is considered the principal mediator in this pathological process. However, the detailed mechanisms involved are unclear. In this study, we attempted to explore the mechanisms involved in LPS-induced cardiomyocyte apoptosis. We found that LPS stimulation inhibited microRNA (miR)-499 expression and thereby upregulated the expression of *SOX6* and *PDCD4* in neonatal rat cardiomyocytes. We demonstrate that *SOX6* and *PDCD4* are target genes of miR-499, and they enhance LPS-induced cardiomyocyte apoptosis by activating the BCL-2 family pathway. The apoptosis process enhanced by overexpression of *SOX6* or *PDCD4*, was rescued by the cardiac-abundant miR-499. Overexpression of miR-499 protected the cardiomyocytes against LPS-induced apoptosis. In brief, our results demonstrate the existence of a miR-499-*SOX6*/*PDCD4*-BCL-2 family pathway in cardiomyocytes in response to LPS stimulation.

**Electronic supplementary material:**

The online version of this article (doi:10.1007/s10495-015-1201-6) contains supplementary material, which is available to authorized users.

## Introduction

Sepsis-induced myocardial functional disorder is one of the main predictors of morbidity and mortality of sepsis [1]; apoptosis is one of the major contributors to the pathophysiology of sepsis [2]. Mediators of sepsis such as lipopolysaccharide (LPS), a gram-negative bacterial cell wall component, triggers apoptosis in cardiac myocytes by promoting the secretion of cytokines such as tumor necrosis factor α (TNF-α), interleukin-6 (IL-6), IL-10, and interferon (IFN)-γ [3].

The *SOX* (sex-determining region [SRY]-related HMG box) genes were initially identified based on their homology to the HMG box (DNA-binding domain) that is highly similar to that of the SRY protein. The *SOX* family comprises 20 genes classified into eight groups [4]. *SOX6* belongs to the Sox D family, and plays important roles in vertebrate development [5–7]. It also induces apoptosis in esophageal squamous cell carcinoma (ESCC) [8, 9]. Previously, we demonstrated that *SOX6* promoted apoptosis at the late stage of P19CL6 cell cardiac differentiation [10]. However, whether *Sox6* is involved in LPS-stimulated cardiomyocyte apoptosis is unclear.

MicroRNAs (miRNAs) exert remarkable effects in diverse apoptosis mechanisms involving *SOX6*. MiR-16 inhibited apoptosis of ESCC cells by downregulating *RECK* and *SOX6* [9]; miR-208 [8] and miR-155 [11] promoted ESCC or hepatocellular carcinoma cell proliferation by targeting *SOX6*. MiRNAs participate in many physiological and pathophysiological processes of the heart, such as cardiac contraction and morphogenesis, myocardial infarction, and heart failure [12–14]. In the myocardium of rats with acute myocardial infarction, the expression of some miRNAs was altered, including cardiac-abundant miRNAs such as miR-1, miR-133, miR-208, and miR-499 [15–17]. Our previous research demonstrated that miR-499 inhibited apoptosis during cardiac differentiation of P19CL6 cells [10]. MiR-499, which is specifically expressed in skeletal muscle and the heart, was first described in 2005 [18]. Its high expression in the heart indicates that it might participate in some of its physiological and pathophysiological processes. Indeed, it was regulated and functioned differentially in heart development [6, 19, 20]. Whether miR-499 participates in LPS-induced apoptosis is unknown, as is the possible mechanism involved.

In addition, one miRNA may target several genes; one gene may be regulated by more than one miRNA. MiR-499 regulates apoptosis through multi-gene targeting. Previously, we discovered that *PDCD4* (programmed cell death 4) was a target of miR-499 in the regulation of hydrogen peroxide (H_2_O_2_)-induced apoptosis [21], where *PDCD4* is upregulated during apoptosis [22]. Subsequently, a number of reports have indicated that it acts as a tumor suppressor and a potential target for anti-cancer therapies [23]. *PDCD4* is also involved in the inflammatory response [24, 25] and differentiation [26, 27]. MiR-21 blocked pancreatic β-cell death [28] and miR-183 suppressed apoptosis in esophageal cancer cells [29] by targeting *PDCD4*. Currently, whether *PDCD4* can be controlled by cardiac-abundant miR-499 in LPS-stimulated cardiomyocyte apoptosis is unclear.

In this study, we investigated whether *SOX6* and *PDCD4*, under common control by miR-499, participate in LPS-induced cardiomyocyte apoptosis. LPS stimulation upregulated *SOX6* and *PDCD4* expression and enhanced cardiomyocyte apoptosis by suppressing miR-499.

## Materials and methods

### Cell culture and chemical reagents

Neonatal rat ventricular myocytes were prepared from 1- or 2-day-old Sprague–Dawley rats as previously described [30]. The cells were cultured in Dulbecco’s modified Eagle’s medium (DMEM; Gibco, Grand Island, NY, USA) supplemented with 15 % fetal bovine serum (FBS) for 36 h, then the culture medium was changed to serum-free medium and the cells were cultured for another 12 h before further experiments. H9c2 cells (ATCC^®^ CRL-1446™, Manassas, VA, USA) and HeLa cells (ATCC^®^ CCL-2™) were maintained in high-glucose DMEM supplemented with 10 % FBS, penicillin (100 U/mL), and streptomycin (100 U/mL). LPS (L4391-1MG) was purchased from Sigma-Aldrich (St. Louis, MO, USA) and dissolved in phosphate-buffered saline (PBS).

### Plasmid constructs and oligonucleotides

pCMV-SPORT-*Pdcd4* (containing the full-length coding sequence of *Pdcd4*) was a gift from Dr. Iwata Ozaki (Health Administration Center, Department of Internal Medicine, Saga Medical School, Saga University). pGL3-*Pdcd4*-3′-UTR was kindly provided by Dr. Giridhar Mudduluru (Department of Experimental Surgery Mannheim/Molecular Oncology of Solid Tumors, Deutsches Krebsforschungszentrum and University Heidelberg). pMIR-REPORT-*Pdcd4*-3′-UTR was constructed by cloning the 3′-untranslated region (3′-UTR) of *PDCD4* from pGL3-*Pdcd4*-3′-UTR into pMIR-REPORT. pcDNA3.1-*Sox6* (containing the full-length coding sequence of *SOX6*) was provided by Dr. Veronique Lefebvre (Case Western Reserve University). pMIR-REPORT-*Sox6*-3′-UTR and its mutant construct have been described previously [10]. Small interfering RNAs (siRNAs) targeting *PDCD4* and *SOX6* (Table 1) were commercially synthesized by Sigma-Aldrich and GeneChem (Shanghai, China), respectively. A scrambled 22-nucleotide (nt) miRNA (negative control [NC]), miR-499 Mimic and miR-499 Inhibitor were obtained from RiboBio (Guangzhou, China).

**Table 1:** siRNAs used for transfection

Gene	Sequence (5′–3′)
*Pdcd4*	Sense: GUCUAAAGGU GGAAAGCGUd TdT
	Anti-sense: ACGCUUUCCA CCUUUAGACd TdT
*Sox6*	Sense: CACUUGUCAGUACCAUUCATT
	Anti-sense: UGAAUGGUACUGACAAGUGTT

### Luciferase assays

HeLa cells were plated in 24-well plates at 5 × 10^4^ cells/well 24 h before transfection. *PDCD4* and *SOX6* luciferase plasmids (400 ng) and 20 ng control *Renilla* vector were cotransfected with transfection reagent (Lipofectamine^®^ 2000; Invitrogen, Carlsbad, CA, USA). Lysates were collected 48 h after transfection, and luciferase activity was measured in triplicate using a dual luciferase assay (Vigorous, Beijing, China).

### Western blotting analysis

Total protein extracts were obtained with lysis buffer (150 mM NaCl, 10 mM Tris [pH 7.2], 5 mM EDTA, 0.1 % sodium dodecyl sulfate [SDS], 1 % sodium deoxycholate, 1 % Triton X-100) containing protease inhibitor cocktail (Sigma-Aldrich). Proteins were separated by electrophoresis on 8–15 % SDS–polyacrylamide gels, transferred to nitrocellulose membranes, and incubated with the corresponding primary antibodies. PDCD4 (sc-27123), BAD (sc-8044), BAX (sc-493), BID (sc-6538), and BCL-xL (sc-8392) antibodies were purchased from Santa Cruz Biotechnology (Santa Cruz, CA, USA); SOX6 (ab64946) antibody was purchased from Abcam (Hong Kong, China); α-actinin (A7811) was from Sigma-Aldrich. The membranes were also probed for mouse glyceraldehyde-3-phosphate dehydrogenase (GAPDH) as a loading control. The blots were next incubated with peroxidase-conjugated immunoglobulin G secondary antibody and were developed using an enhanced chemiluminescence kit (Millipore, Billerica, MA, USA).

### Quantitative real-time PCR

Total RNA was isolated with TRIzol (Invitrogen), and 2 μg total RNA was reverse-transcribed with random primers for complementary DNA (cDNA) synthesis. The cDNA was used for PCR using specific primers. Transcript levels were normalized to 18S rRNA. The primers are listed in Table 2. Each value represents the average of at least three independent experiments.

**Table 2:** Primers used for quantitative real-time RT-PCR

Primers	Sequence (5′–3′)	Product size (bp)
*Pdcd4*	F: TGCCCGTGTT GGCAGTGTC R: TGGCCCACCAACTGTGGTGC	190
*Sox6*	F: CCCCTCTGAACATGGTGGTGGC R: TGAGACTGCCCCTGCCGAGT	145
*Bad*	F: AAGTCCGATCCCGGAATCC R: GCTCACTCGGCTCAAACTCT	106
*Bax*	F: AGTGATGGACGGGTCCGGGG R: GGCGGCTGCTCCAAGGTCAG	156
*Bid*	F: TCTGAGGTCAGCAACGGTTC R: CTCTTGGCGAGTACAGCCAG	95
*Bcl*-*xL*	F: CTGACGCCCTTCACCGCGAG R: CAAAGGCATCCCAGCCTCCGT	235

### TUNEL assay and annexin V/PI staining

Cardiomyocyte apoptosis was measured using terminal deoxynucleotide transferase dUTP nick end labeling (TUNEL) (Roche Life Science, Indianapolis, IN, USA) according to the manufacturer’s protocol. Cells cultured on coverslips in 24-well plates were fixed in 4 % paraformaldehyde. The number of TUNEL-positive cells was counted under a fluorescence microscope. For flow cytometry analysis, briefly, cultured cells were harvested by trypsinization and washed with PBS. Cells (1 × 10^6^) from each sample were processed for annexin V/propidium iodide (PI) apoptosis detection (Dojindo, Kumamoto, Japan) according to the manufacturer’s instructions.

### Statistical analysis

The data are reported as the mean ± standard deviation. Comparisons were analyzed using Student’s *t* test or ANOVA. *p* < 0.05 was considered to indicate statistical significance.

## Results

### MiR-499 levels were downregulated in response to LPS stimulation

**Fig. 1:**
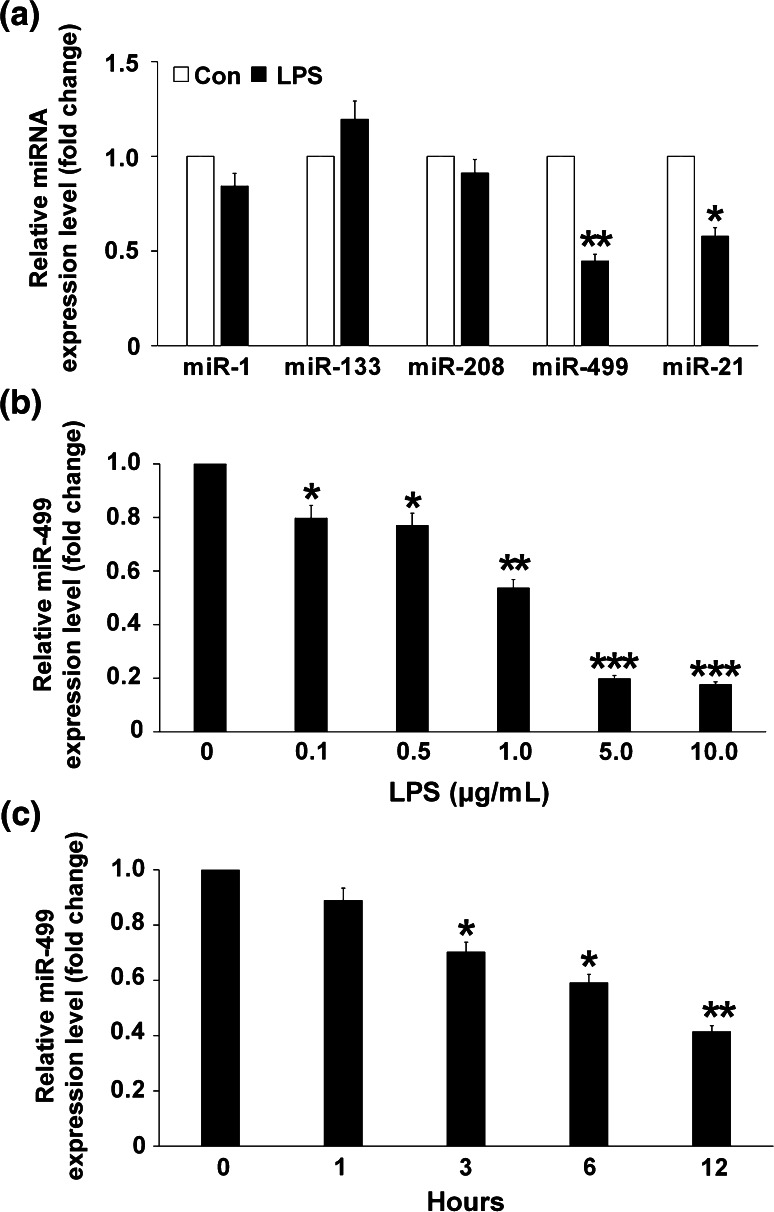
MiR-499 was downregulated in response to LPS stimulation. **a** Cardiac-abundant miRNA level in neonatal rat cardiomyocytes exposed to 1 μg/mL LPS at 6 h. Cardiomyocytes not treated with LPS were used as the negative control (con). **b** MiR-499 level in neonatal rat cardiomyocytes exposed to LPS for 6 h. Cardiomyocytes not treated with LPS (0 μg/mL) were used as the negative control. **c** MiR-499 level in neonatal rat cardiomyocytes exposed to varying durations of 1 μg/mL LPS. Cardiomyocytes not treated with LPS (0 h) were used as the negative control. Data represent the results of three independent experiments. *p < 0.05, **p < 0.01, ***p < 0.001 compared with negative control

Cardiac-abundant miRNAs such as miR-1, miR-133, miR-208, and miR-499 regulate diverse aspects of cardiac function, including cardiomyocyte proliferation, differentiation, contractility, and stress responsiveness. To examine their roles in cardiac cell response to LPS stimulation, we treated cardiomyocytes with 1 μg/mL LPS. Short exposure (6 h) to LPS decreased miR-499 expression, but did not alter expression of the other miRNAs significantly. A known LPS-responsive miRNA, miR-21 [25], was used as the positive control, and showed decreased expression (Fig. 1a). The LPS-induced expression of miR-499 in the cardiomyocytes was concentration- and time-dependent (Fig. 1b, c).

### MiR-499 protected cardiomyocytes from LPS-induced apoptosis

**Fig. 2:**
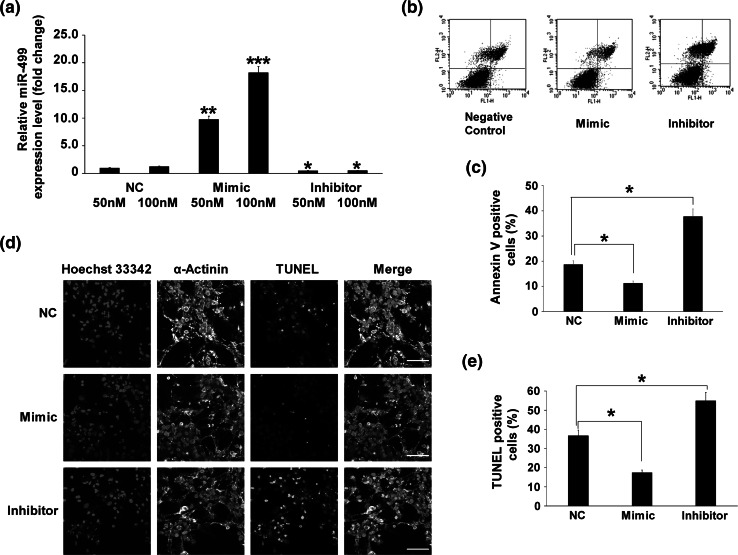
MiR-499 protected cardiomyocytes from LPS-induced apoptosis. **a** MiR-499 level in cardiomyocytes after 48-h transfection with miR-499 Mimic or Inhibitor. **b** Representative flow cytometry images of annexin V/PI-stained cardiomyocytes treated with miR-499 Mimic or Inhibitor, and then exposed to 1 μg/mL LPS for 6 h. **c** Quantitative results for annexin V-positive cardiomyocytes from three independent experiments. **d** Representative images of TUNEL-stained cardiomyocytes treated with miR-499 Mimic or Inhibitor, and then exposed to LPS as above. *Red* TUNEL-stained positive nuclei, *green* α-actinin antibody staining, *blue* Hoechst33342-stained nuclei, *scale bars* 50 μm. **e** Quantitative results for apoptotic cells from three independent experiments. A scrambled 22-nt miRNA was used as the negative control (NC). *p < 0.05, **p < 0.01, ***p < 0.001 compared with negative control (NC) (Color figure online)

To study whether miR-499 participates in LPS-induced apoptosis, we established overexpression and knockdown systems by transfecting cardiomyocytes with miR-499 Mimic (chemically synthesized fragments with the same sequence as miR-499 that enhance endogenous miR-499 function) or with miR-499 Inhibitor (chemically synthesized fragments with reversed complementary sequence to miR-499 that weaken endogenous miR-499 effects), a scrambled 22-nt miRNA was used as the NC (Fig. 2a). Flow cytometry (Fig. 2b, c) and TUNEL (Fig. 2d, e) were employed to verify apoptotic cell numbers. The Mimic-treated cardiomyocytes were less susceptible to 1 μg/mL LPS treated for 6 h, with a lower apoptosis rate than the NC-treated cardiomyocytes; while apoptosis was potentiated in Inhibitor-treated cardiomyocytes compared to the NC-treated group.

Taken together, these results indicate that the miR-499 level is important for maintaining cardiac cell survival in response to LPS stimulation.

### SOX6 and PDCD4 participated in LPS-induced cardiomyocyte apoptosis

**Fig. 3:**
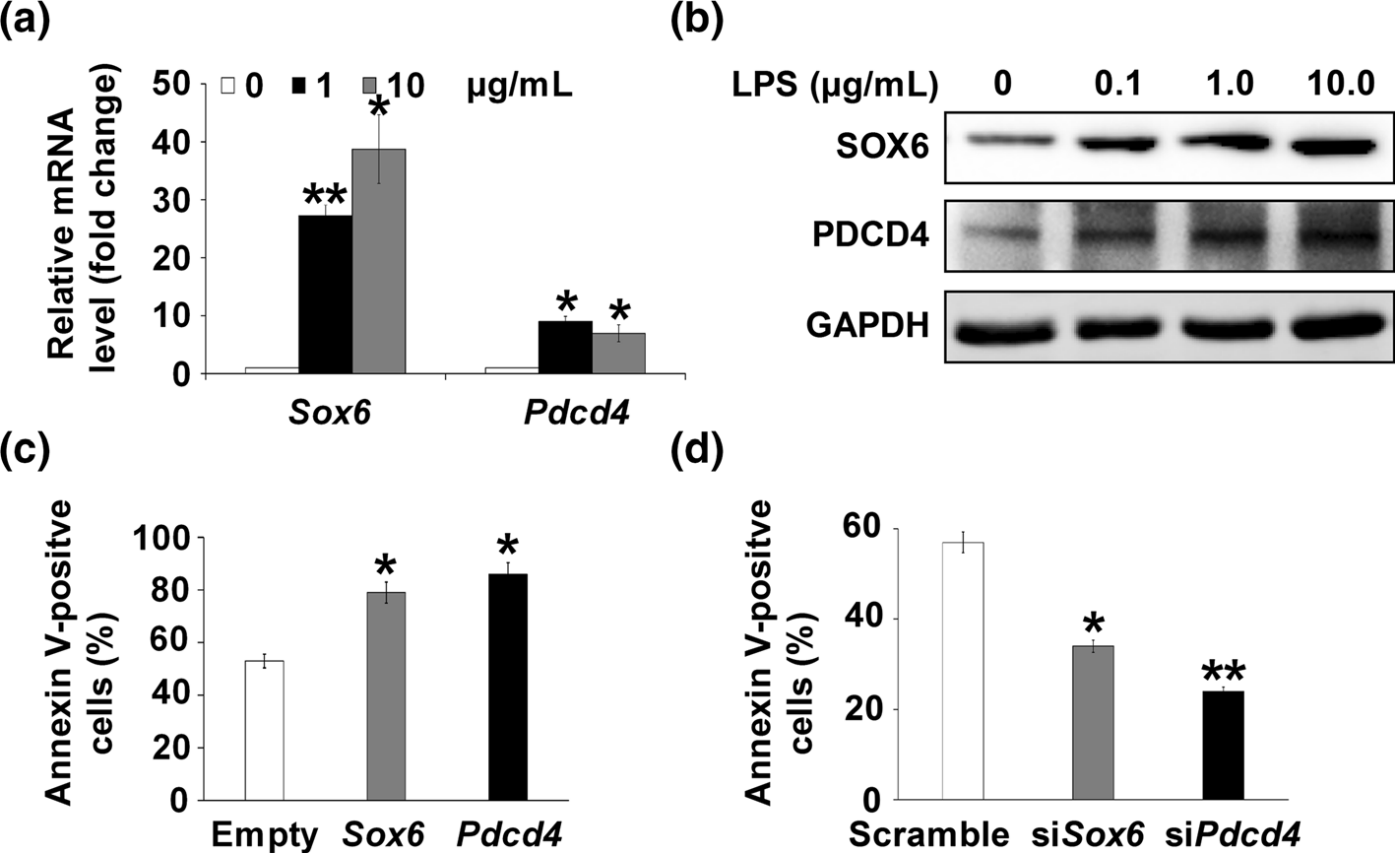
SOX6 and PDCD4 are involved in LPS-induced cardiac cell apoptosis. **a** *SOX6* and *PDCD4* mRNA level in cardiomyocytes exposed to LPS. Cardiomyocytes not treated with LPS (0 μg/mL) were used as the negative control. **b** SOX6 and PDCD4 protein level in LPS-treated cardiomyocytes. **c**, **d** Quantitative flow cytometry results for annexin V/PI-stained cells transfected with *SOX6* or *PDCD4* plasmid (**c**) or with *SOX6* siRNA or *PDCD4* siRNA (**d**), respectively, and then exposed to 1 μg/mL LPS for 6 h. The vector plasmid pcDNA3 (Empty) or scrambled siRNA were used as the negative control. *p < 0.05, **p < 0.01 compared with negative control. Data represent the results of three independent experiments

To elucidate the molecular mechanisms by which miR-499 regulates apoptosis, we focused on *SOX6* and *PDCD4*, which play important roles in cardiomyocyte differentiation of P19CL6 cells [10] and in protecting adult cardiomyocytes against oxidative stress [21], respectively. *SOX6* and *PDCD4* expression was first examined to determine whether it is related to LPS-induced cardiomyocyte apoptosis. The results show that *SOX6* and *PDCD4* expression at both mRNA and protein levels was upregulated in response to 1 μg/mL LPS stimulation (Figs. 3a, b, S1). To explore how *SOX6* and *PDCD4* are integrated into the cell death program triggered by LPS, the H9c2 cell line was selected for further study. H9c2 is a cardiac myoblast cell line, similar to primary cardiomyocytes [31]. In response to LPS stimulation, *SOX6* and *PDCD4* overexpression enhanced the rate of apoptosis from 50 % to almost 80 % (Fig. 3c); knockdown of either *SOX6* or *PDCD4* decreased the rate of apoptosis from approximately 50–25 % (Fig. 3d). However, without LPS stimulation, the overexpression of *SOX6* and *PDCD4* only slightly increased the apoptosis rate from 6 to 9 % or 13 % respectively (Fig. S2a), while knockdown of either *SOX6* or *PDCD4* did not affect the apoptosis rate (Fig. S2b), indicating that the effect of *SOX6* and *PDCD4* is more significant under the circumstance of LPS-induced damage.

Taken together, our observations suggest that *SOX6* and *PDCD4* promote LPS-mediated apoptosis.

### SOX6 and PDCD4 were targets of miR-499 in LPS-induced apoptosis

**Fig. 4:**
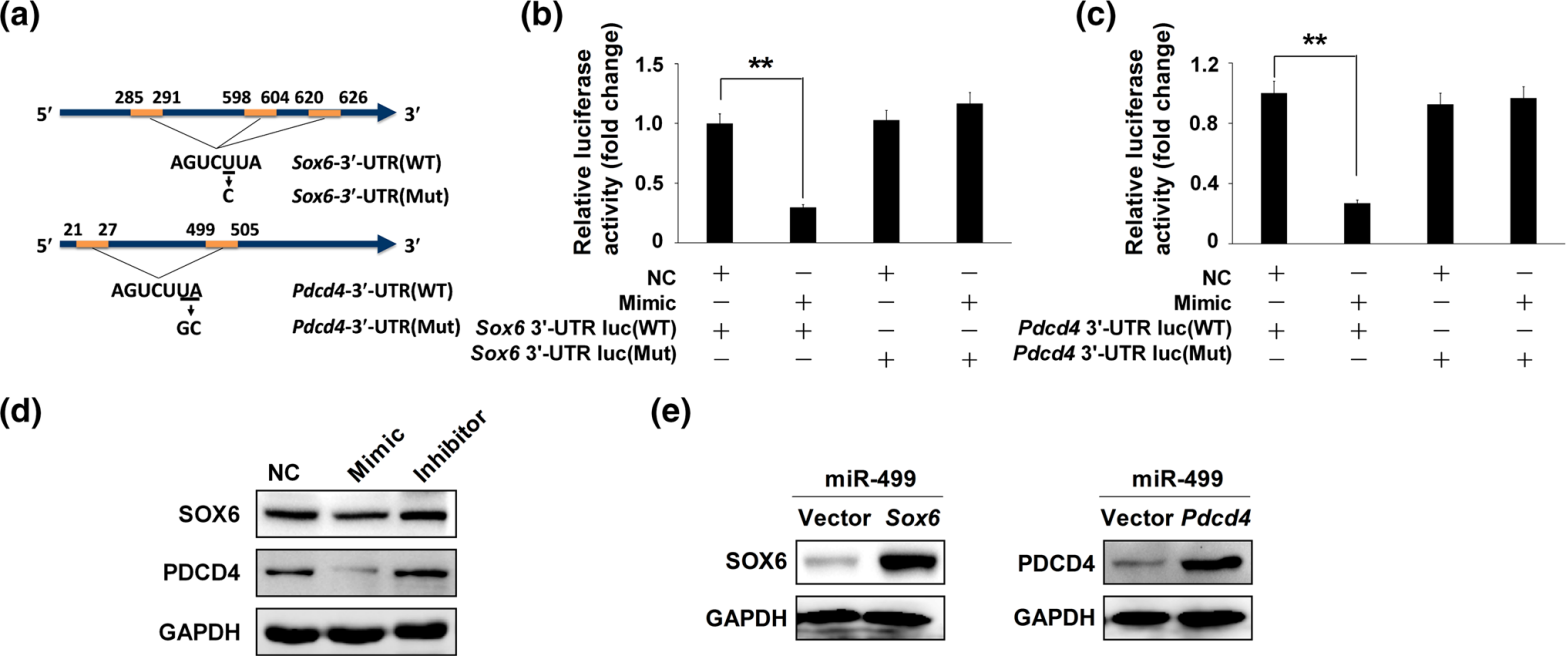
*SOX6* and *PDCD4* are targets of miR-499. **a** Schematic illustration indicates the seed sequences on the 3′-UTR of *SOX6* or *PDCD4*, which are potential target genes of miR-499. The mutant binding sites are underlined. **b**, **c** Luciferase analysis of the effect of miR-499 on its potential targets. Luciferase activity was measured in HeLa cells cotransfected with miR-499 and *SOX6* 3′-UTR luciferase reporter (luc) (wild type [WT]) or *SOX6* 3′-UTR luc (mutant [Mut]) (**b**), *PDCD4* 3′-UTR luc (WT) or *PDCD4* 3′-UTR luc (Mut) (**c**). **d** SOX6 and PDCD4 protein level in cardiomyocytes treated with miR-499 NC, Mimic, or Inhibitor. GAPDH was used as the internal control. **e** Expression of *SOX6* (*left*) and *PDCD4* (*right*) in H9c2 cells cotransfected with miR-499 Mimic in combination with *SOX6* or *PDCD4* plasmid, respectively. *p < 0.05, **p < 0.01 compared with negative control. Data represent the results of three independent experiments

To investigate whether miR-499 affects the expression of the endogenous target genes, we analyzed the *SOX6* and *PDCD4* sequences and generated luciferase reporters with the 3′-UTR of *SOX6* and *PDCD4*, and the constructs contained a mutated segment of *SOX6* (seed sequence AGUCUUA was mutated to AGUCCUA) and *PDCD4* (seed sequence AGUCUUA was mutated to AGUCUGC), respectively (Fig. 4a). As shown in Fig. 4b, c, miR-499 Mimic reduced luciferase activity significantly compared to the NC, whereas no effect was observed with the mutant constructs. This effect was specific because there was no change in luciferase reporter activity when the NC was cotransfected with each reporter construct. Meanwhile, Western blot analysis indicated that miR-499 Mimic attenuated *SOX6* and *PDCD4* expression, whereas miR-499 Inhibitor elevated it (Fig. 4d). These results suggest that *SOX6* and *PDCD4* both are direct miR-499 targets.

To investigate whether miR-499 inhibited cardiomyocyte apoptosis by suppressing *SOX6* and *PDCD4*, we performed a rescue experiment in miR-499-treated cells by transfecting pcDNA3.1-*Sox6* or pCMV-SPORT-*Pdcd4* plasmids. Because pcDNA3.1-*Sox6* and pCMV-SPORT-*Pdcd4* constructs do not contain 3′-UTR in which the miR-499 binding sites locate, they were not targeted by miR-499. Therefore, Western blot showed that SOX6 and PDCD4 protein level was highly upregulated (Fig. 4e).

### miR-499 activated the SOX6 and PDCD4 pathways

**Fig. 5:**
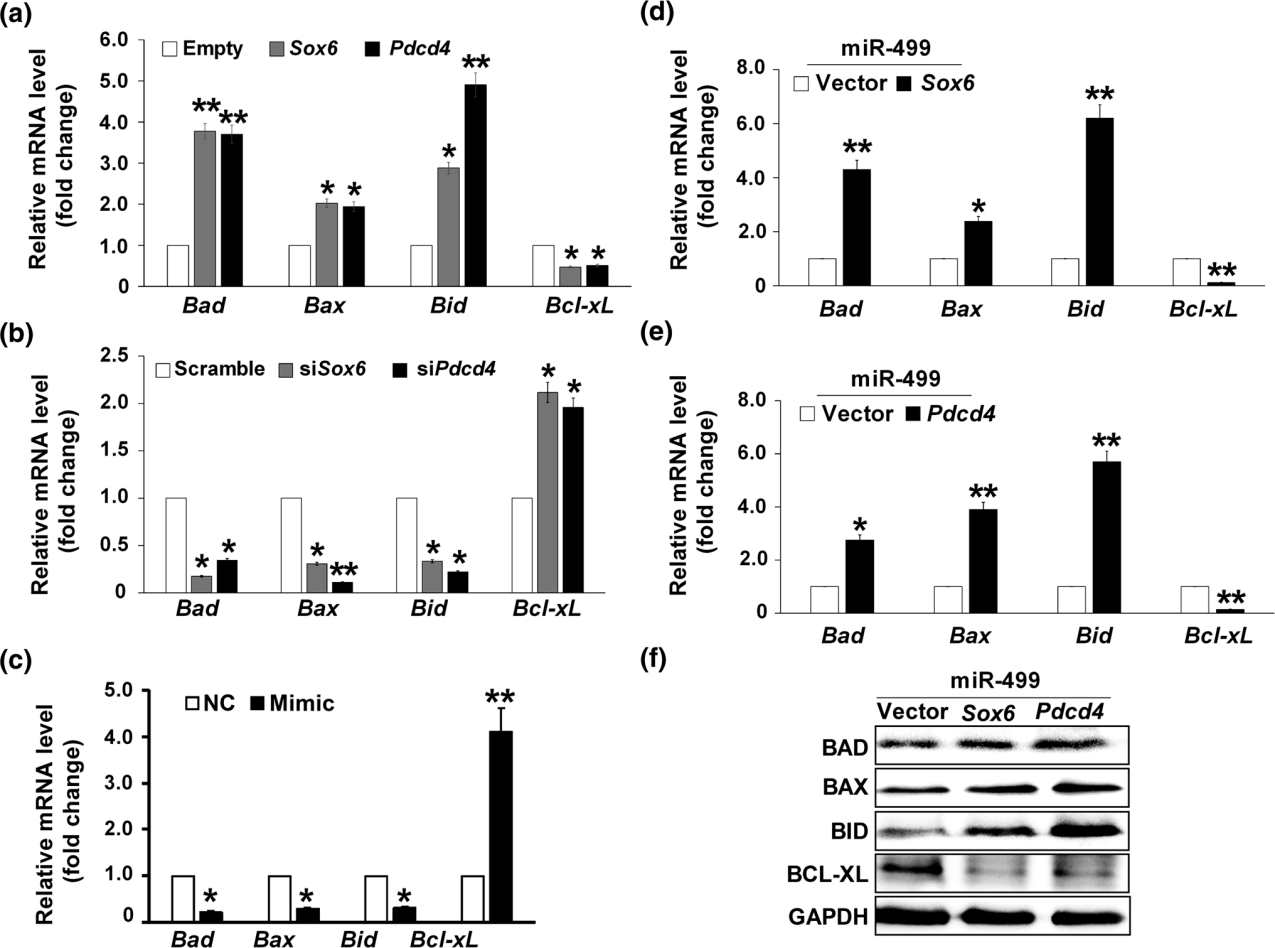
LPS inhibits miR-499 to activate the SOX6 and PDCD4 pathways. **a**, **b** mRNA Level of *BAD*, *BAX*, *BID*, and *BCL*-*XL* in H9c2 cells transfected with *SOX6* or *PDCD4* plasmid (**a**) or with *SOX6* or *PDCD4* siRNA (**b**), respectively. **c** Endogenous mRNA level of *BAD*, *BAX*, *BID*, and *BCL*-*XL* in response to 6-h stimulation with 1 μg/mL LPS in H9c2 cells transfected with miR-499 NC, Mimic. **d**, **e** mRNA Level of *BAD*, *BAX*, *BID*, and *BCL*-*XL* in H9c2 cells cotransfected with miR-499 Mimic in combination with *SOX6* (**d**) or *PDCD4* (**e**) plasmid, respectively. **f** BAD, BAX, BID, and BCL-XL expression in H9c2 cells cotransfected with miR-499 Mimic in combination with *SOX6* or *PDCD4* overexpression plasmid, respectively, and then exposed to 1 μg/mL LPS for 6 h. GAPDH was used as the internal control. *p < 0.05, **p < 0.01 compared with negative control. Data represent the results of three independent experiments

BCL-2 family members are major regulators of mitochondrial integrity and mitochondria-initiated caspase activation. The BCL-2 family has both anti-apoptotic and pro-apoptotic members, and act as the downstream key factors of many apoptosis mediators [32]. To investigate how SOX6 and PDCD4 regulate LPS-induced cardiomyocyte apoptosis, pcDNA3.1-*Sox6* and pCMV-SPORT-*Pdcd4* overexpression plasmids as well as their specific siRNAs were used to transfect H9c2 cells. Western blotting indicated that the overexpression plasmids and specific siRNAs successfully enhanced or inhibited *SOX6* and *PDCD4* expression (Fig. S3). In H9c2 cells transfected with *SOX6* or *PDCD4* overexpressing constructs, the mRNA level of the pro-apoptotic genes (*BAD*, *BAX*, *BID*) was upregulated, whereas that of the anti-apoptotic gene *BCL*-*XL* was downregulated with 1 μg/mL LPS for 6 h (Fig. 5a). But without LPS stimulation, the transfection of *SOX6* or *PDCD4* did not affect these genes’ expression significantly (Fig. S4). In contrast, the mRNA level of *BAD*, *BAX*, and *BID* was significantly decreased in the *SOX6* or *PDCD4* knockdown system, whereas that of *BCL*-*XL* was increased (Fig. 5b).

In LPS-treated rat cardiomyocytes, miR-499 overexpression inhibited *Bad*, *Bax*, and *Bid* mRNA level, and promoted *Bcl*-*xL* level (Fig. 5c); meanwhile, the expression of these genes was not affected significantly by miR-499 without LPS treatment (data not shown). In LPS-treated rat cardiomyocytes, the cotransfection of *Sox6* or *Pdcd4* with miR-499 reversed the miR-499-mediated cardiac protective effects, which included upregulation of *Bad*, *Bax*, and *Bid* mRNA/protein, and downregulating *Bcl*-*xL* mRNA/protein (Fig. 5d–f), indicating the existence of an miR-499-*Sox6*/*Pdcd4*-apoptosis pathway.

## Discussion

Cardiomyocyte apoptosis is one of the major pathogenic factors in heart diseases, including septic cardiomyopathy. Bacterial endotoxins, such as LPS, are considered the principal cause of myocardial dysfunction. However, the mechanisms involved are unclear. In this study, we attempted to explore the mechanisms involved in LPS-induced cardiomyocyte apoptosis. We discovered that LPS stimulation reduced miR-499 expression, thereby relieving the inhibitory effect on its target genes *SOX6* and *PDCD4*, which then activated the BCL-2 family pathway to participate in LPS-induced apoptosis.

SOX6 is a key regulator in cell differentiation and organ development. It also plays important roles in proliferation and apoptosis during stem cell differentiation or cancer progression. In hepatocellular carcinoma, SOX6 activates *p21WAF1/CIP1* (p21) expression in a p53-dependent manner, and miR-155 targeting of *SOX6* facilitated cell proliferation [11]. In pancreatic β-cells, SOX6 inhibited *cyclin D1* promoter activity and negatively regulated cell proliferation by interacting with histone deacetylase 1 (HDAC1) and β-catenin [33]. *SOX6* suppression also caused retinoic acid-dependent apoptosis and blocked neuronal differentiation in the early stages of P19 cell neuronal differentiation [34]. However, the involvement of SOX6 in LPS-induced cardiomyocyte apoptosis has not been reported. In our previous research, we found that SOX6, as a repressor of *cyclin D1*, arrested cardiomyocyte proliferation and facilitated cell cycle exit [10]. The present study is the first demonstration, to our knowledge, that *SOX6* expression is elevated in LPS-treated cardiomyocytes in a time- and dose-dependent manner, and the upregulated SOX6 enhances LPS-induced apoptosis.

We also found that *PDCD4* was upregulated in LPS-treated cardiomyocytes. PDCD4 is a tumor repressor, suppressing tumor growth through different mechanisms [35]. PDCD4 also plays an important role in various inflammatory diseases. *PDCD4*-deficient cells were significantly less sensitive to apoptosis, and *PDCD4* overexpression in β-TC-6 cells increased their susceptibility to TNF-α plus IFN-γ-induced apoptosis [28]. PDCD4 promoted activation of the transcription factor nuclear factor-κB (NF-κB) and suppressed IL-10; *Pdcd4*-deficient mice were less susceptible to LPS-induced death, suggesting PDCD4 is a proinflammatory factor [25]. However, other reports showed that *PDCD4* deficiency increased TNF-α protein expression in LPS-treated RAW264.7 macrophages, indicating that PDCD4 is an anti-inflammatory factor [36, 37]. Therefore, the exact role of PDCD4 in inflammatory diseases remains to be investigated. It should be noted that the effect of LPS stimulation on *PDCD4* expression differs between reports. In primary bone marrow-derived macrophages and RAW264.7 macrophages, *PDCD4* was upregulated at 1 h after LPS treatment and gradually decreased at 6 h [24]. Another report showed that *PDCD4* remained at a high level at 8 h after LPS stimulation in human peripheral blood mononuclear cells [25]. It is easy to understand that dynamic feedback is involved in the response to various stimuli; any difference could lead to different feedback. Nevertheless, we found that *PDCD4* was upregulated in LPS-stimulated cardiomyocytes.

To understand how *SOX6* and *PDCD4* expression is regulated in LPS-treated cardiomyocytes, we detected the expression of a series of miRNAs. MiR-499 and miR-21 were downregulated in LPS-induced cells in a dose- and time-dependent manner, while there was no significant change to miR-1, miR-133, and miR-208 expression. A growing amount of evidence demonstrates that miRNAs can regulate apoptosis [38]. Thirteen miRNAs, including miR-21, were upregulated in early-stage dilated cardiomyopathy, whereas 11 miRNAs including miR-499 were downregulated [39]. MiR-499 was also downregulated in cardiomyocytes exposed to anoxia [40]. In our previous work, we showed that miR-499 and miR-21 were upregulated in H_2_O_2_-induced cardiomyocyte apoptosis [21]. Hence, miRNA expression is correlated with various diseases, stimuli, cells, and detection time [41]. Several reports demonstrated that transient transfection of miR-1 and miR-499 reduced proliferation and enhanced differentiation into cardiomyocytes in human cardiac progenitor cells and embryonic stem cells [6]. In a transgenic mouse model, elevated miR-499 levels affected *Egr1* and *Fos*, the immediate early genes in the response to cardiac stress [31]. Additionally, in rat myocardial infarction area induced by anoxia and ischemia, miR-499 inhibited cardiomyocyte apoptosis through regulation of mitochondrial dynamics by targeting calcineurin and dynamin-related protein-1 [40].

In the present study, LPS stimulation suppressed miR-499 expression, which led to elevation of the cardiomyocyte apoptosis rate. Our experiments also showed that the BCL-2 family is involved in miR-499-*SOX6*-*PDCD4* apoptotic regulation. MiR-499 inhibited the expression of pro-apoptotic genes and upregulated expression of the anti-apoptotic gene *BCL*-*XL*. *SOX6* and *PDCD4* reversed these effects, suggesting that miR-499 regulates *BAD*, *BAX*, *BID*, and *BCL*-*XL* expression by inhibiting *SOX6* and *PDCD4*, thereby playing a key role in LPS-induced cardiomyocyte apoptosis. To investigate how LPS downregulates miR-499, sequence analysis of the promoter region of *MYH7B*, the host gene of miR-499, was performed. The result showed that it contains one NF-κB binding site, which is conserved among rats, mice, and humans. NF-κB is activated by LPS-induced TNF-α [2]. However, when we transfected the luciferase reporter construct containing a 1000-bp region of the *MYH7B* regulatory fragment upstream of the translational start site into H9c2 cells that were then treated with LPS, no significant change in luciferase activity was observed after LPS treatment (data not shown). It should be noticed that we used H9c2 cells for the most experiments to demonstrate SOX6 and PDCD4 as the targets of miR-499. Although this cell line is different from primary neonatal myocytes, it is commonly used as a cardiomyocyte model for mechanism investigation [42, 43]. However, further investigation is required to clarify the upstream regulation mechanism of miR-499. In summary, LPS stimulation relieved the inhibitory effect of miR-499 on its target genes *SOX6* and *PDCD4*, which enhanced LPS-induced cardiomyocyte apoptosis through the BCL-2 family members. Overexpression of miR-499 protected cardiomyocytes against LPS-induced apoptosis.

## Electronic supplementary material

The relative expression of *SOX6* or *PDCD4* to *GAPDH* in LPS-treated H9c2 cells, *p < 0.05, **p < 0.01 compared with 0 μg/mL LPS group. Data represent the results of three independent experiments. Supplementary material 1 (TIFF 58 kb)

Quantitative flow cytometry results for annexin V/PI-stained H9c2 cells transfected with *Sox6* or *Pdcd4* plasmid (**a**) or with *Sox6* siRNA or *Pdcd4* siRNA (**b**), respectively, without LPS treatment. The vector plasmid pcDNA3 (Empty) or scrambled siRNA were used as the negative control. *p < 0.05, compared with negative control. Data represent the results of three independent experiments. Supplementary material 2 (TIFF 110 kb)

SOX6 and PDCD4 protein level in H9c2 cells transfected with *Sox6* and *Pdcd4* overexpression plasmids (OE) or siRNA against *Sox6* and *Pdcd4* (#1 to #3 represent three different siRNAs for each gene. #1 for *Sox6* and #2 for *Pdcd4* were used in the subsequent experiments. GAPDH was used as the internal control. Supplementary material 3 (TIFF 515 kb)

mRNA Level of *Bad*, *Bax*, *Bid*, and *Bcl*-*xl* in H9c2 cells transfected with *Sox6* or *Pdcd4* plasmid respectively. The empty vector (Empty) was used as the negative control. Data represent the results of three independent experiments. Supplementary material 4 (TIFF 50 kb)
