# Supplementary figures and images for: SOX6 and PDCD4 enhance cardiomyocyte apoptosis through LPS-induced miR-499 inhibition
Source: Apoptosis. 2015 Dec 10;21:174–83. doi: 10.1007/s10495-015-1201-6 (PMC4712245; doi:10.1007/s10495-015-1201-6)

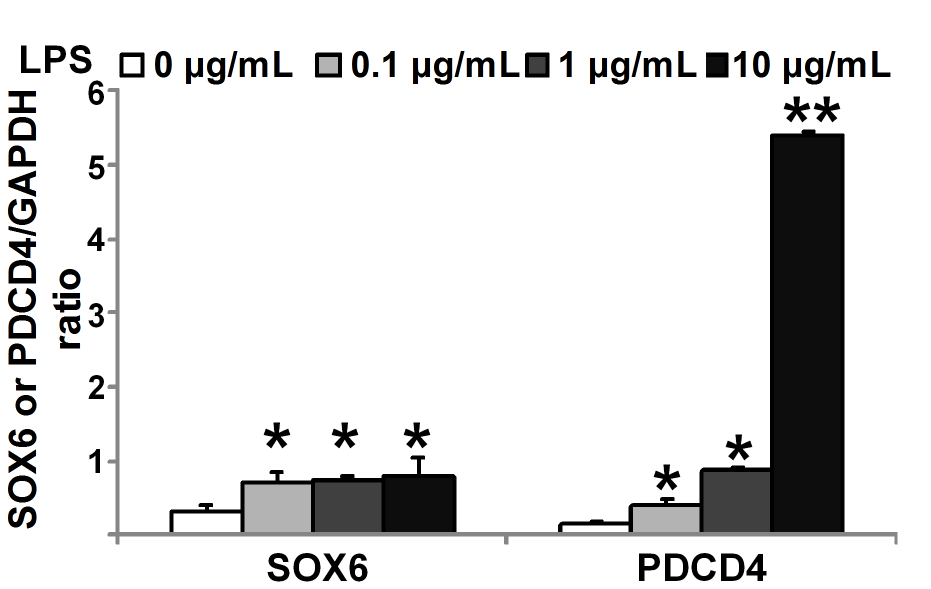

Supplement: Supplementary file 1 — The relative expression of SOX6 or PDCD4 to GAPDH in LPS-treated H9c2 cells, *p < 0.05, **p < 0.01 compared with 0 μg/mL LPS group. Data represent the results of three independent experiments. Supplementary material 1 (TIFF 58 kb) [file 10495_2015_1201_MOESM1_ESM.tif]

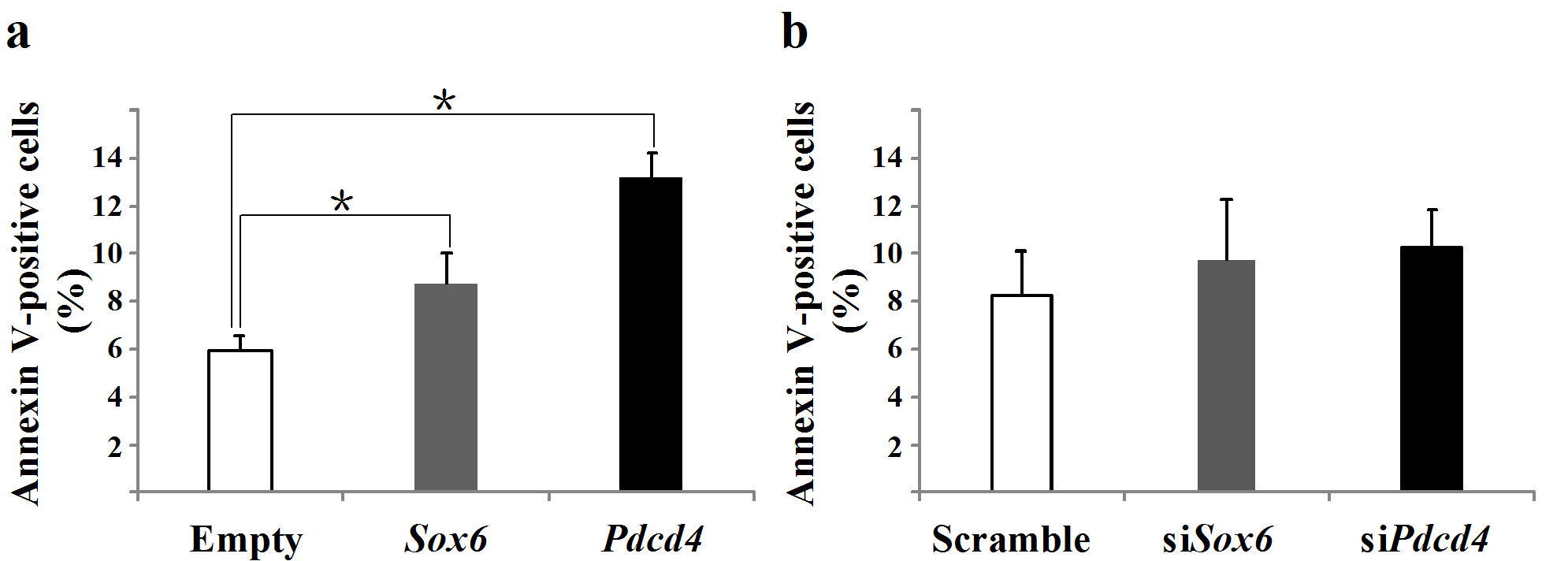

Supplement: Supplementary file 2 — Quantitative flow cytometry results for annexin V/PI-stained H9c2 cells transfected with Sox6 or Pdcd4 plasmid (a) or with Sox6 siRNA or Pdcd4 siRNA (b), respectively, without LPS treatment. The vector plasmid pcDNA3 (Empty) or scrambled siRNA were used as the negative control. *p < 0.05, compared with negative control. Data represent the results of three independent experiments. Supplementary material 2 (TIFF 110 kb) [file 10495_2015_1201_MOESM2_ESM.tif]

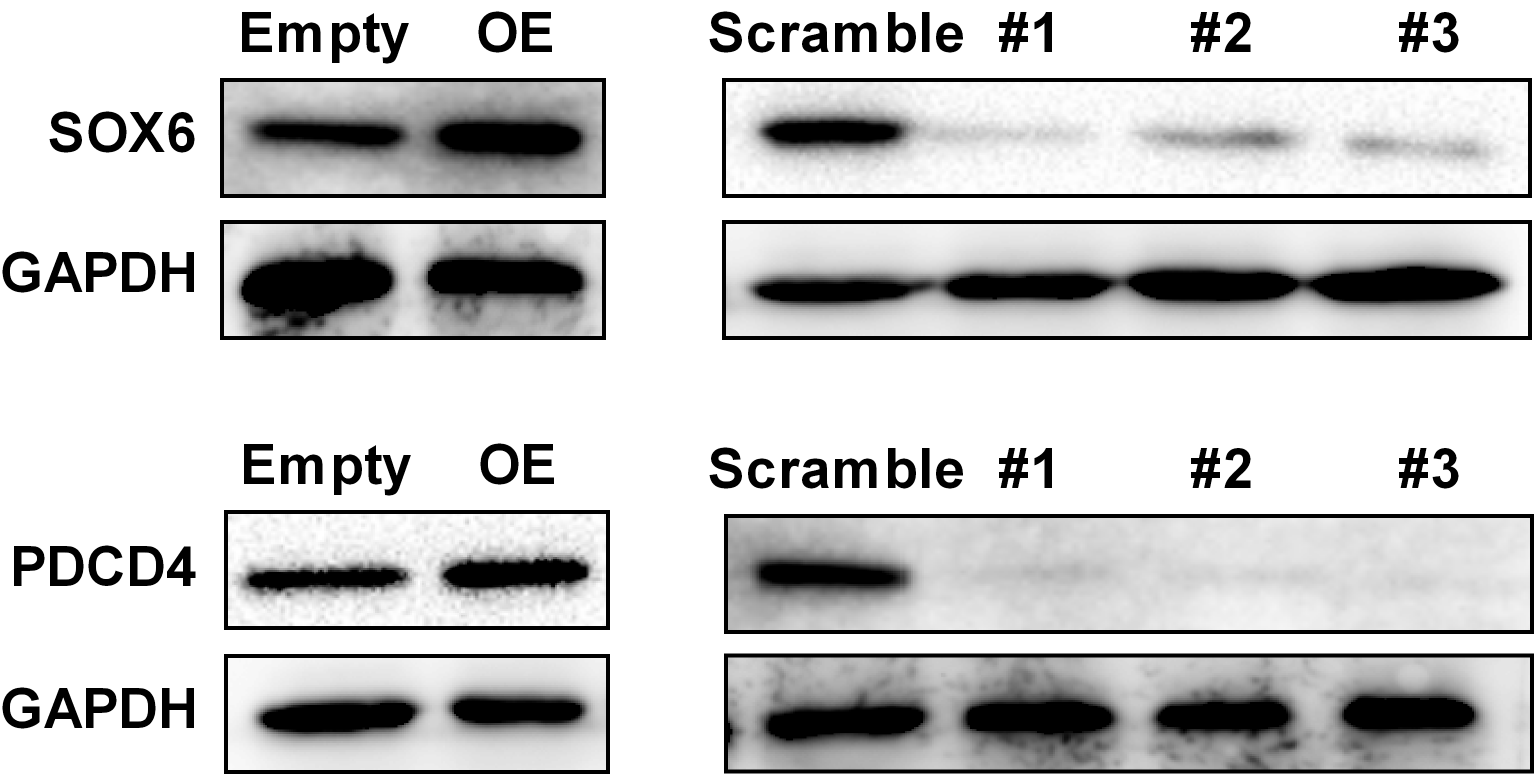

Supplement: Supplementary file 3 — SOX6 and PDCD4 protein level in H9c2 cells transfected with Sox6 and Pdcd4 overexpression plasmids (OE) or siRNA against Sox6 and Pdcd4 (#1 to #3 represent three different siRNAs for each gene. #1 for Sox6 and #2 for Pdcd4 were used in the subsequent experiments. GAPDH was used as the internal control. Supplementary material 3 (TIFF 515 kb) [file 10495_2015_1201_MOESM3_ESM.tif]

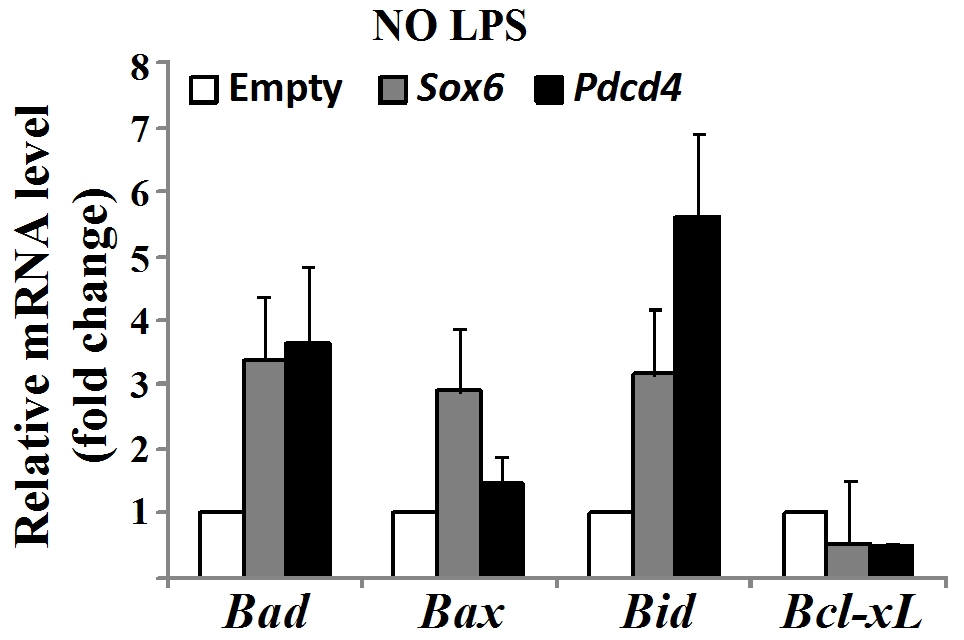

Supplement: Supplementary file 4 — mRNA Level of Bad, Bax, Bid, and Bcl-xl in H9c2 cells transfected with Sox6 or Pdcd4 plasmid respectively. The empty vector (Empty) was used as the negative control. Data represent the results of three independent experiments. Supplementary material 4 (TIFF 50 kb) [file 10495_2015_1201_MOESM4_ESM.tif]
